# Physiological and morphological investigation of Arctic grayling (*Thymallus arcticus*) gill filaments with high salinity exposure and recovery

**DOI:** 10.1093/conphys/cox040

**Published:** 2017-06-28

**Authors:** Salvatore D. Blair, Derrick Matheson, Greg G. Goss

**Affiliations:** 1 Department of Biological Sciences, University of Alberta, Edmonton, Alberta, Canada T6G 2E9

**Keywords:** hydraulic fracturing, hypersaline, ILCM, osmoregulation, salmonid

## Abstract

Freshwater environments are at risk of increasing salinity due to multiple anthropogenic forces including current oil and gas extraction practices that result in large volumes of hypersaline water. Unintentional releases of hypersaline water into freshwater environments act as an osmoregulatory stressor to many aquatic organisms including native salmonids like the Arctic grayling (*Thymallus arcticus*). Compared to more euryhaline salmonids, Arctic grayling have a reduced salinity tolerance and develop an elevated interlamellar cell mass (ILCM) in response to salinity exposure (17 ppt). In this study, we described the gill morphology and cell types characterizing the ICLM. Further, we investigated whether Arctic grayling could recover in freshwater following a short-term (<48 h) salinity exposure. Arctic grayling were exposed to 17 ppt saline water for 12, 24 and 48 h. Following the 24 and 48 h salinity exposure, Arctic grayling were returned to freshwater for 24 h to assess their ability to recover from, and reverse, the osmotic disturbances. Physiological serum [Na^+^], [Cl^–^] and total osmolality were significantly elevated and progressively increased at 12, 24 and 48 h salinity exposures. The 24 h post-exposure recovery period resulted in Arctic grayling serum ion concentrations and total osmolality returning to near normal levels. Similar recovery patterns were observed in the salinity-induced ILCM, which developed as early as 12 h of exposure to 17 ppt, and then reverted to control levels following 24 h in freshwater. Gill histology indicates an increased number of apically located mucous cells in the interlamellar space following salinity exposure of Arctic grayling. The scanning electron microscopy and transmission electron microscopy data show the presence of granule containing eosinophil-like cells infiltrating the ILCM suggesting a salinity-induced immune response by the Arctic grayling.

## Teaser

Arctic grayling develop an interlamellar cell mass (ILCM) in their gill filaments when exposed to a half-strength seawater saline exposure but can recover if a freshwater environment is re-established within 48 h post saline water exposure. Microscopy analysis reveals that ILCM development is associated with a salinity-induced immunologic response.

## Introduction

The salmonid, Arctic grayling (*Thymallus arcticus*) is the only *Thymallus* species, of the monogeneric sub-family Thymallinae, that is native to North America. Historically, *T. arcticus* range extended west of Hudson Bay to Alaska, across the northern halves of British Columbia, Alberta, Saskatchewan and Manitoba, with populations found in Michigan and Montana (Stamford and Taylor, 2004). Across their Holarctic range, this species has a secure global conservation status with a large total population. However, there are local native populations in North America that range from extirpated to vulnerable conservation status (Page and Burr, 2011; NatureServe, 2017). The southern-most populations in Alberta and Montana, have been declining over the past century, with the complete extirpation of Michigan populations. The exact reasons for this decline are unknown but likely include habitat fragmentation, overfishing, climate change and watershed effects brought on by the oil and gas industry (Walker, 2005).

Recent conservation and research efforts have been aimed at assessing the environmental challenges encountered by this threatened species. Landscape-level research provides evidence for negative effects of habitat fragmentation due to road construction and culverts on river populations of fish. There are current practices being implemented to reconnect fish to their spawning areas and reduce negative effects of culverts on fish passage (Park *et al.*, 2008; MacPherson *et al.*, 2012). Overfishing effects have been directly addressed in Alberta by the implementation of a zero-harvest regulation for grayling province wide (AEP, 2015). Furthermore, temperature effects on grayling are being investigated and will help to elucidate temperature-sensitive areas and the populations at risk. The habitat of the southern population of Arctic grayling overlaps with some of the most significant oil and gas deposits in North America and unlocking these reserves relies on relatively newer technologies of horizontal drilling and hydraulic fracturing. These industrial practices result in large volumes of hypersaline water, and uncontrolled releases from pipeline or truck spills have been documented (Alessi *et al.*, 2016; Maloney *et al.*, 2017). Upon incidental release into waterbodies, these hypersaline waters can result in severe osmoregulatory challenges for exposed freshwater aquatic organisms (He *et al.*, 2016; Blewett *et al.*, 2017).

The ability to osmoregulate is in direct relation to the function of the gill or branchial epithelium of the fish (Evans *et al.*, 2005). In general, teleost fish gills are composed of two sets of four gill arches. Each arch is composed of many laterally branching filaments, which contain an afferent and efferent blood vessel that branch individually to the many folds of the epithelial surface or lamellae, and allow for a countercurrent exchange of gasses and ions with the water (Evans *et al.*, 2005). The branchial epithelium is made up of pavement cells, mucous cells and mitochondrial rich cells (MR cells). The MR cells are responsible for the bulk of ion transport and are found on the trailing edge of the filament as well as near the base of the lamellae facing the interlamellar space where water flow occurs (Perry, 1997; Evans *et al.*, 2005).

Osmoregulation is critical for homoeostatic balance and is hindered by osmotic disturbances such as a sudden change in environmental salinity. Hydraulic fracturing practices result in large volumes of hypersaline water, up to 10× that of seawater (300 ppt) (Allen, 2008). Spills of this produced water are not uncommon, with 51 incidents of varying volumes of saline water releases reported from January 2016 to June 2016 in Alberta alone (AER, 2016). Some of these hypersaline releases affect standing or flowing water, and under these circumstances osmotic stress on aquatic organisms would occur. As with any toxicant, the degree of damage is proportional to both volume introduced and length of time of the release.

With the exception of a few cases of individuals from Arctic coastal river populations being captured in brackish estuary waters (<18 ppt) (see review/comment: Heim *et al.*, 2016), Arctic grayling are considered stenohaline freshwater salmonids. Recently, we have demonstrated that land-locked Artic grayling are more salinity-sensitive than another, often co-occurring, salmonid species the rainbow trout. Even a short-term exposure to a salinity level of 50% seawater or 17 ppt (a concentration which would be the result of a ~20-fold dilution of commonly produced hypersaline-produced water), exceeds the 96 h salinity tolerance threshold and osmoregulatory compensation ability of Arctic grayling while rainbow trout are able to tolerate this level of hypersaline exposure (Blair *et al.*, 2016). It was suggested that mortality was due to the grayling’s non-anadromous behaviour and an associated inability to up-regulate the necessary seawater isoform sodium-potassium ATPase alpha1b (*nkaα1b*). Importantly, exposure to 17 ppt saline water resulted in gill remodelling and the appearance of an ILCM in the grayling that was not present in rainbow trout under the same treatments (Blair *et al.*, 2016). Ionoregulatory and osmoregulatory perturbations have been shown to alter the ILCM in a variety of fish: hypoxia-induced changes in ILCM in goldfish and crucian carp (Sollid *et al.*, 2003; Sollid and Nilsson, 2006; Nilsson *et al.*, 2012); temperature variations in goldfish, crucian carp (Sollid *et al.*, 2005; Mitrovic and Perry, 2009) and killifish (McBryan *et al.*, 2016); high environmental ammonia on crucian carp (Sinha *et al.*, 2014); air exposure and salinity on killifish (Ong *et al.*, 2007; LeBlanc *et al.*, 2010; Turko *et al.*, 2011) and increases to ILCM of brook trout exposed to aluminium (Mueller *et al.*, 1991). We hypothesized that the ILCM appearance in the Arctic grayling in response to salinity could be a protective mechanism limiting the uptake of salts (Na^+^/Cl^–^) under hypertonic environments.

This study intended to address the question whether Arctic grayling exposed to a higher salinity for a short-term (24 or 48 h) can recover and reduce the resultant ILCM. We further investigated the general gill morphology with a goal to identify the cell types present in both the normal gill epithelium and ILCM of Arctic grayling.

## Methods

### Animal collection

Arctic grayling (18.1 ± 0.4 cm, 49.7 ± 3.9 hg, means ± SE) were collected in July 2015 from a tributary found in the Athabasca River watershed in collaboration with Alberta Environment and Parks (AEP) using conventional angling methods (fly-fishing). The grayling were transferred in an Alberta fish transportation truck to the University of Alberta, where they were held in a bio-secure aquatics facility, in a 600 l circular tank supplied with flow-through dechlorinated Edmonton city tap water (10°C) and constantly aerated. Fish were acclimated for 3 months prior to experimentation. Arctic grayling were fed a mixture of *Artemia* and *Mysis* shrimp, every other day.

### Experimental tanks

Two identical systems (freshwater and a saline) were used for the exposure experiments. Each system consisted of three (60 l) in-line tanks, a top header tank, an exposure tank (housed fish) and a sump tank, which re-circulated 180 l of water maintained at 10°C with an attached chiller unit. Both systems were initially supplied with dechlorinated Edmonton city tap water, which was aerated with air stones placed in each exposure tank. Dissolved oxygen levels were maintained above 10 mg/l. The saline system water was made up to 17 ppt (50% seawater) with the addition of a soluble salt mixture (Instant Ocean, Blacksburg, VA, USA). This salinity concentration was chosen based on our previous research revealing sub-lethal effects at this salinity. When necessary, salinity levels were maintained by adding salts and/or freshwater following measurement using a handheld digital salinity probe (YSI Model 85; Yellow Springs, OH, USA).

### Salinity exposure and sampling

Arctic grayling were fasted for four days prior to experimentation. Fish (*n* = 5 for each treatment) were transferred to either 17 ppt saline water and held for 12, 24 and 48 h, or to the freshwater system for 24 h (control) before lethal sampling. For investigations into the Arctic grayling’s ability to recover following the 24 and 48 h acute saline exposure, fish were transferred back to a freshwater environment where they were allowed to recover for an additional 24 h before undergoing lethal sampling.

Fish were anesthetized with a lethal dose of MS-222 (200 mg/l buffered with 400 mg/l NaHCO_3_), weighed and measured (fork length), followed by immediate blood and tissue sampling. Blood was drawn from caudal puncture using non-heparinized syringes, injected into 1.5 ml Eppendorf tubes and centrifuged (2 min, 12 000 × *g*). Serum was separated and frozen at −80°C for future analysis. The second and third gill arches were excised from each side of the fish and processed for light microscopy, scanning electron microscopy (SEM) and transmission electron microscopy (TEM).

### Physiological serum levels

Arctic grayling serum from each of the six treatments was thawed and analysed for total osmolarity (mOsm), sodium [Na^+^] and chloride [Cl^–^]. Following essential dilution in ultrapure water, analysis of serum [Na^+^] was performed using an atomic absorption spectrophotometer (Varian 220 FS). Serum [Cl^–^] was measured via digital chloridometer (Labconco, Kansas City, MO, USA). Total serum osmolarity analysis was performed using a freezing point depression osmometer (Micro Osmette; Precision Systems). Statistical analysis was performed with one-way ANOVA, followed by Tukey’s multiple comparisons test (Graphpad Systems: Prism 6).

### Light microscopy

For consistency purposes, the right third gill arch from each grayling was excised and fixed in a 4% paraformaldehyde (PFA) in phosphate-buffered saline (PBS) (in mmol/l: 137 NaCl, 2.7 KCl, 4.3 Na_2_HPO_4_ and 1.4 NaH_2_PO_4_; pH 7.4) for 24 h at 4°C. Gill tissue processing followed a standard histological protocol consisting of washes in PBS, followed by a dehydration series of increasing ethanol concentrations (0 to 70% EtOH), followed by paraffin wax embedding. Gills were mounted in wax blocks and sectioned serially with a microtome at 7 μm thickness. The sections were mounted on Superfrost plus microscope slides (Thermo Fisher Scientific, Rockford, IL, USA). Five slides per gill were obtained containing 3–4 serial sections on each slide. Slides were stained with either periodic acid–Schiff (PAS) stain for localization of mucous cells, or hematoxylin and eosin for general histological analysis and viewed on a compound light microscope (Zeiss AXIO Scope.A1, Germany) fitted with a digital camera (Optronics, CA, USA). A single slide per fish was randomly selected and digital images were captured (PictureFrame Software Ver, 2.3). To maintain ILCM measurement consistency across treatments, three filaments from the most central region of the gill face of each fish were selected under 20× magnification, and subsequently 10 lamellae lengths and interlamellar cell mass measurements per filament were taken using ImageJ software (National Institutes of Health). As previously described (Ong *et al.*, 2007; Blair *et al.*, 2016), ratios of interlamellar cell mass to lamellae height were calculated for each fish (*n* = 5) per treatment and averages were compared via one-way ANOVA, with Tukey’s Multiple Comparisons test (Graphpad Systems: Prism 6). PAS-stained mucous cells were quantified in a similar manner by calculating the average number of mucous cells per individual ILCM region. Total cells from 10 adjacent ILCM regions of three different filaments were counted per fish (*n* = 4) for the following treatments: FW, 48 h exposure and 48 h exposure + recovery. Statistical analysis on each treatment was performed as above via one-way ANOVA, with Tukey’s Multiple Comparisons test (Graphpad Systems: Prism 6).

### Electron microscopy

For SEM and TEM analysis, gill filaments were dissected from the left third gill arch. For SEM, pairs of filaments were dissected away from the middle of the gill arch, while individual filaments were cut under a dissecting microscope for TEM analysis. Both samples were fixed overnight at room temperature in a buffered 0.15 M sodium cacodylate solution (pH 7.4) containing 2.5% glutaraldehyde and 3% PFA. Filaments underwent three 10-min washes in 0.15 M sodium cacodylate buffer, followed by dehydration in a series of ethanol washes (0–70% EtOH). Following complete dehydration, the SEM filaments were placed in a serial series of increasing concentrations of hexamethyldisilazane and allowed to air dry. SEM filaments were mounted on to SEM stubs and sputter coated with a Au/Pd mixture and observed using scanning electron microscope (XL30, FEI Company, OR, USA) with Scandium 5.0 imaging software.

TEM filaments were immersed for 1 h in 0.15 M sodium cacodylate buffer containing 1% osmium tetroxide, followed by a 0.15 M sodium cacodylate buffer wash step prior to dehydration through the graded ethanol series. TEM filaments were individually embedded into SPURR Resin blocks and sectioned using an ultramicrotome (Reichert-Jung Ultracut_E Ultramicrotome). Thin sections (~80 nm) were placed on copper grids and stained with a uranyl acetate and lead citrate stain. Examination and imaging of sections was conducted with a transmission electron microscope (Philips-FEI, Morgagni 268), equipped with a Gatan Orius CCD camera.

## Results

### Physiological serum analysis

**Figure 1:**
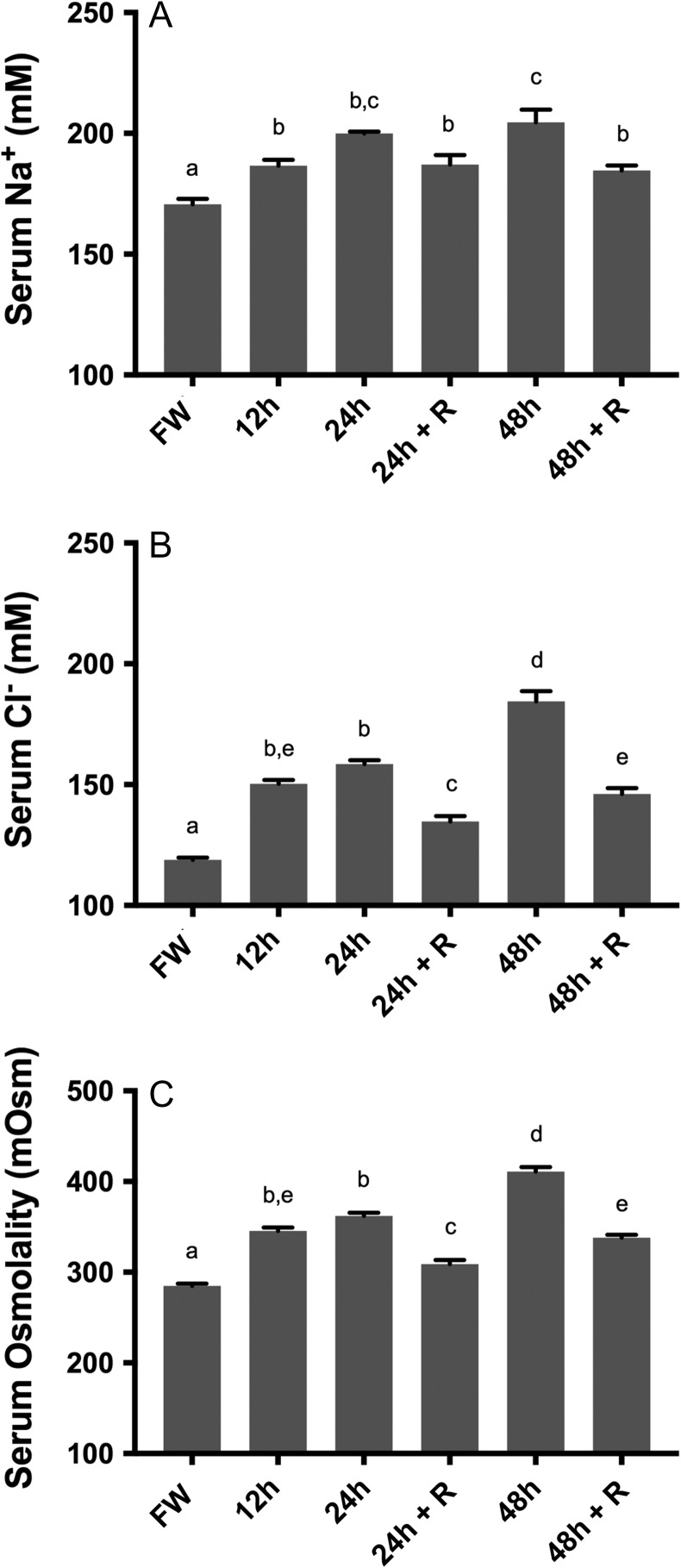
Physiological serum chemistry of Arctic grayling exposed to 17 ppt for 24 and 48 h, and following a 24 h recovery in freshwater following the 17 ppt exposure. (+R) indicates fish allowed to recover in freshwater for 24 h, following exposure to 17 ppt for 24 and 48 h time points. (**A**) Serum sodium [Na^+^], (**B**) serum chloride [Cl^–^], (**C**) total serum osmolality [mOsm]. Data are presented as mean ± SE, while dissimilar letters designate statistically significant differences between groups as demonstrated by one-way ANOVA, with Tukey’s multiple comparisons test (*n* = 5, *P* < 0.05, ANOVA).

Similar to our previous study, serum [Na^+^], serum [Cl^–^] and serum osmolality significantly (*P* < 0.05) and progressively increased when Arctic grayling were exposed to 12, 24 and 48 h of 17 ppt (50% seawater) water. At 48 h of exposure, Arctic grayling serum levels were significantly elevated to 204.54 ± 5.27 mM Na^+^, 184.40 ± 4.29 mM Cl^–^ and 411.0 ± 5.0 mOsm (mean ± SE), an increase of 20%, 55% and 44%, respectively, from freshwater levels (Fig. 1A–C).

Fish that were allowed a 24 h recovery period in freshwater following the exposure to 17 ppt for 24 h showed significant decreases towards pre-exposure values in in serum [Cl^–^], and serum osmolality (*P* < 0.05). Serum [Na^+^] also tended to decrease, albeit non-significantly, in the 24 h + recovery sample. A similar osmotic recovery pattern was observed in fish that were returned to freshwater for 24 h, following exposure to 17 ppt for 48 h. Significant differences were seen between samples from 48 h exposure and the 48 h + recovery samples, with serum [Na^+^] decreasing by 10%, serum [Cl^–^] decreasing by 21% and total serum osmolality decreasing by 18%. Although 24 h recovery in freshwater resulted in significant decreases from both the 24 and 48 h exposure periods, serum ion levels were still significantly elevated above those of fish held in control freshwater (Fig. 1A–C).

### Interlamellar cell mass

**Figure 2:**
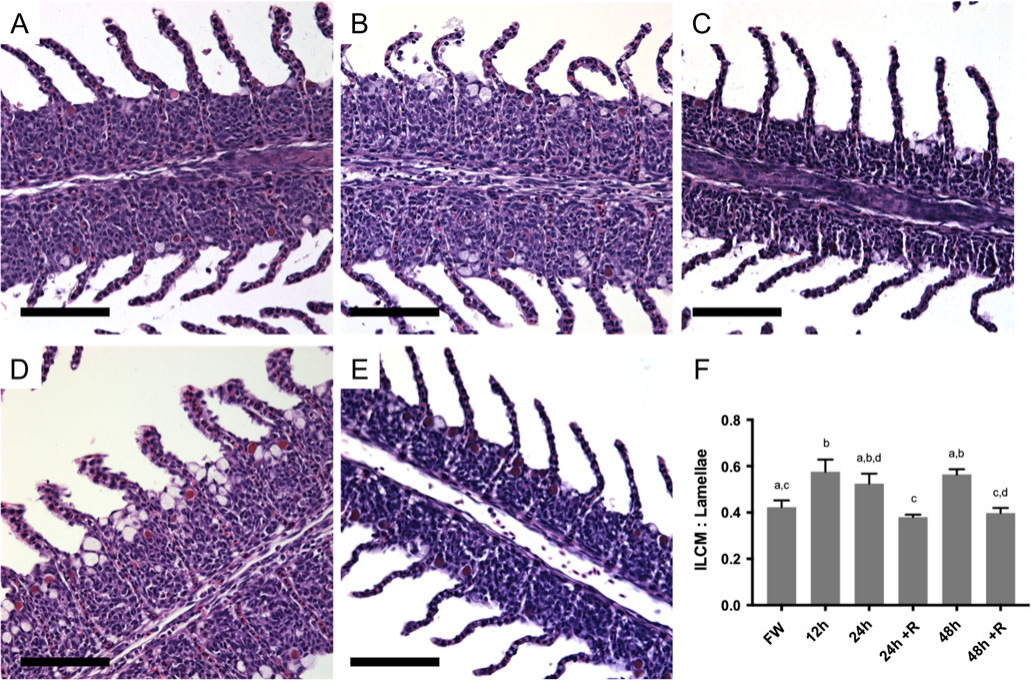
Representative images of haematoxylin and eosin (H&E) stained filaments of Arctic grayling exposed to 17 ppt for 24 and 48 h and those allowed to recover in freshwater for 24 h, demonstrating reduction of ILCM. (**A**) Freshwater control exposure (FW); (**B**) 24 h exposure to 17 ppt with (24 h) elevated ILCM; (**C**) 24 h exposure to 17 ppt + recovery in freshwater with reduced ILCM (24 h +R); (**D**) 48 h exposure to 17 ppt, elevated ILCM (48 h); (**E**) 48 h exposure to 17 ppt + recovery in freshwater with reduced ILCM (48 h +R). Scale bar = 100 μM. (**F**) Height to length ratio of ILCM to lamellae from the various treatment groups. Data are presented as mean ± SE, while dissimilar letters designate statistically significant differences between groups as demonstrated by one-way ANOVA, with Tukey’s multiple comparisons test (*n* = 5, *P* < 0.05, ANOVA).

Measurements of lamellar length and height of interlamellar cell mass were taken at each of the sampling time points (Fig. 2). Compared to freshwater values, significant elevations in ILCM were present just 12 h after exposure to 17 ppt water (*P* < 0.05, one-way ANOVA). The ILCM remained elevated at the 24 and 48 h time points. However, for both the 24 and 48 h exposures, after a 24 h recovery period in freshwater, there were significant reductions in ILCM height (*P* < 0.05, one-way ANOVA) (Fig. 2F). This salinity-induced interlamellar cell mass began to decrease and slough off following 24 h recovery in freshwater (Fig. 2F).

**Figure 3:**
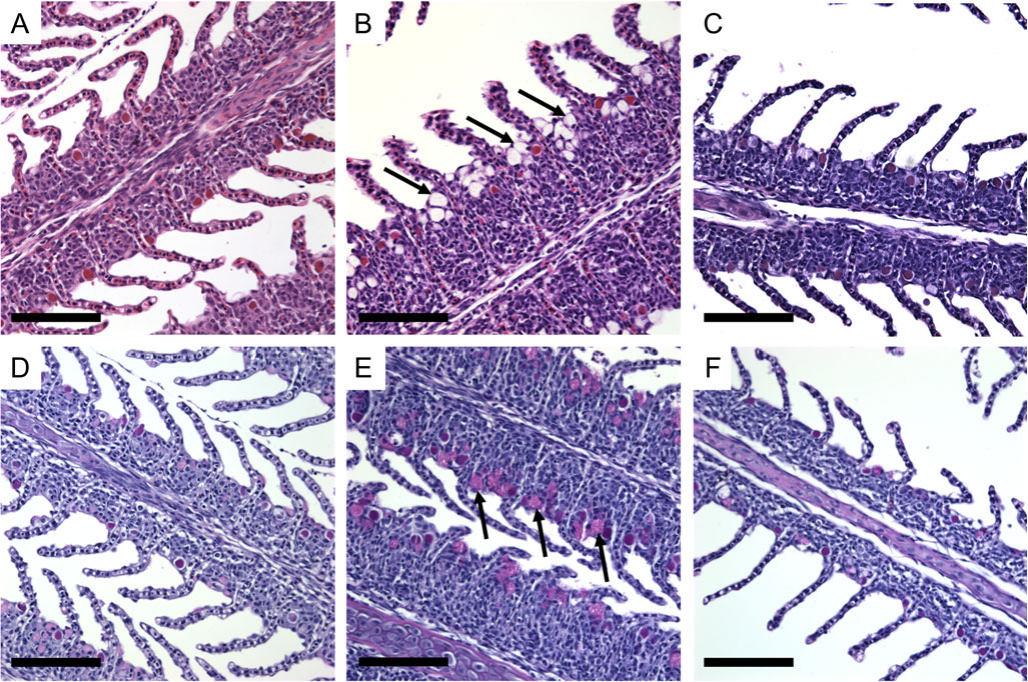
H&E (top panel) and PAS-stained (bottom panel) Arctic grayling gills. Increased number of mucous cells in fish exposed to 17 ppt for 48 h compared to fish held in freshwater and following 24 h recovery post-exposure. Mucous cells (indicated by arrows) appear clear under H&E stain, and stain purple with PAS. (**A,D**) Freshwater; (**B,E**) 48 h exposed; (**C,F**) 48 h exposed + recovery. Scale bar = 100 μM.

**Figure 4:**
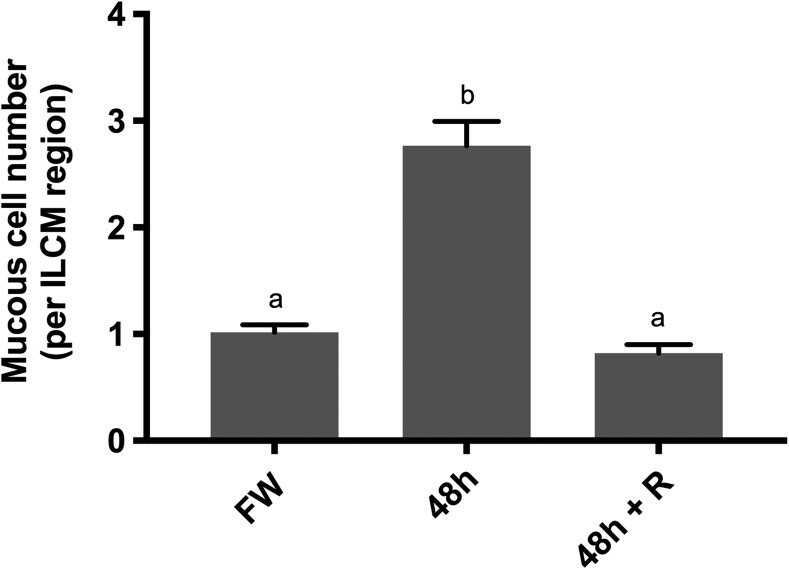
Quantification of mucous cells from Arctic grayling under control freshwater conditions, exposed to 17 ppt for 48 h, and following 24 h recovery post-exposure. Average mucous cells per ILCM region were counted for each treatment. Significant proliferation of mucous cells occurred following exposure to 17 ppt saline water. Following 24 h recovery in freshwater, there is a reduction in mucous cells associated with the retreating ILCM. Data are presented as mean ± SE, while dissimilar letters designate statistically significant differences between groups as demonstrated by one-way ANOVA, with Tukey’s multiple comparisons test (*n* = 4, *P* < 0.0001, ANOVA).

Histological analysis of hematoxylin and eosin stained gill filaments revealed an abundance of mucous-like cells apically located in the ILCM region of fish following salinity exposure compared to those under freshwater control conditions and those following 24 h recovery in freshwater (Fig. 3A–C). Following exposure to 17 ppt, PAS staining confirmed an increase in mucous cells on the apical side of the epithelium associated with the elevated interlamellar cell mass (Fig. 3D–F). These cells were more abundant in 48 h salinity exposed animals (Fig. 3E) than in the freshwater control animals (Fig. 3D) or in the freshwater recovery animals following the initial 48 h exposure to 17 ppt (Fig. 3F). Average mucous cells per ILCM region were calculated as 1.02 ± 0.07 (mean ± SE) under freshwater control conditions; 2.76 ± 0.23 following exposure to 17 ppt for 48 h; and 0.82 ± 0.08 following recovery in freshwater for 24 h (Fig. 4). Quantification of the positively PAS-stained cells confirmed significant proliferation in Arctic grayling ILCM regions when exposed to 17 ppt for 48 h (*P* < 0.0001, *n* = 4, one-way ANOVA).

### Electron microscopy

**Figure 5:**
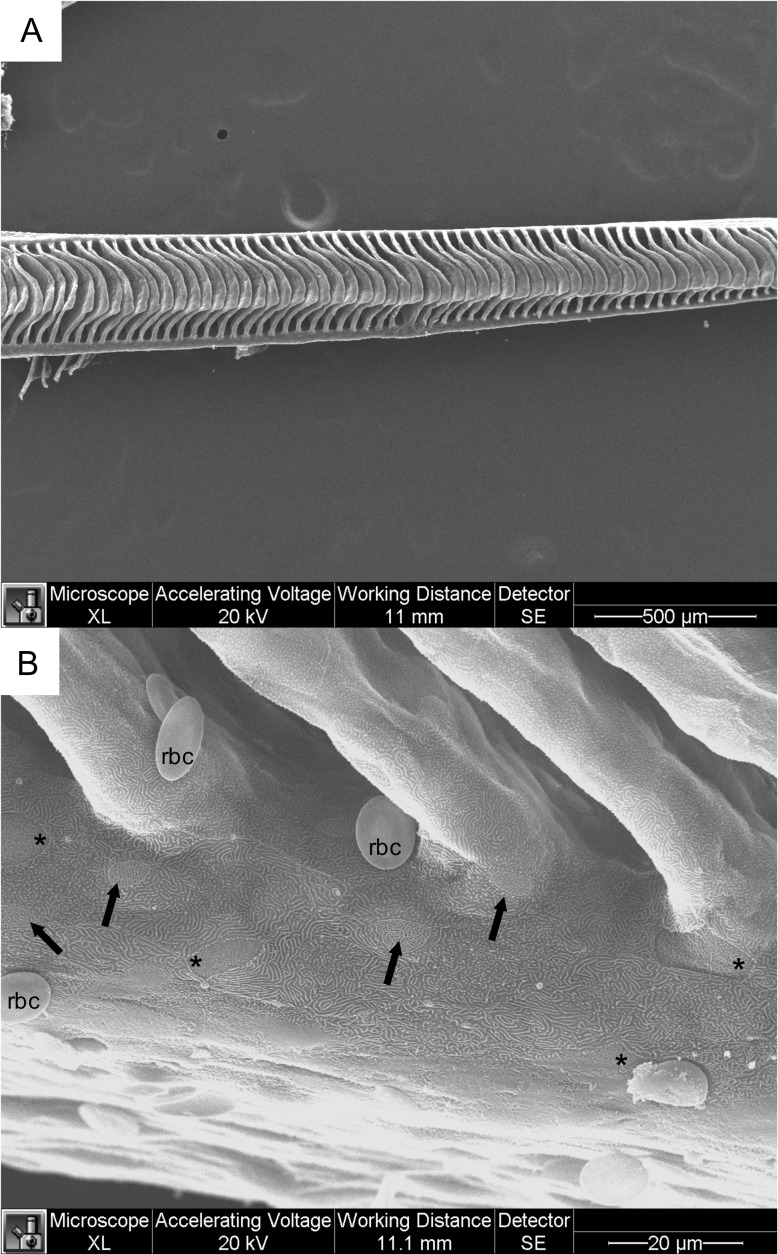
Scanning electron micrograph of rainbow trout gills following 24 h exposure to 17 ppt salinity. (**A**) Single gill filament with exposed protruding lamellae. (**B**) Higher magnification of trailing edge of filament near base of protruding lamellae. Apical surface reveals pavement cells covered with of interconnected uniform microplicae, and two types of MR cells interspersed among pavement cells, one with finger-like projections marked with asterisks (*) and one with tightly packed microvilli forming with a maze-like appearance indicated with arrows (↑).

Morphological comparisons of Arctic grayling branchial epithelia with that of the rainbow trout following 24 h exposure to 17 ppt salinity were performed using SEM and TEM. As described previously for the morphology of the trout gill (Goss *et al.*, 1994; Perry, 1997; Evans *et al.*, 2005) the lamellae of the rainbow trout expanded to the edge of the filament, with a relatively small area of epithelial cells on either side made up both the leading and trailing edge (Fig. 5A). Rainbow trout gill epithelial morphology consisted of a covering layer of pavement cells, with a maze-like formation of interconnected uniform microplicae on their surface. Mitochondrial rich cells with two distinct surface morphologies were commonly found interspersed amongst the pavement cells, one surface characterized by many finger-like microvilli projections, while the other had a tightly packed multicursal labyrinth configuration of microvilli (Fig. 5B).

**Figure 6:**
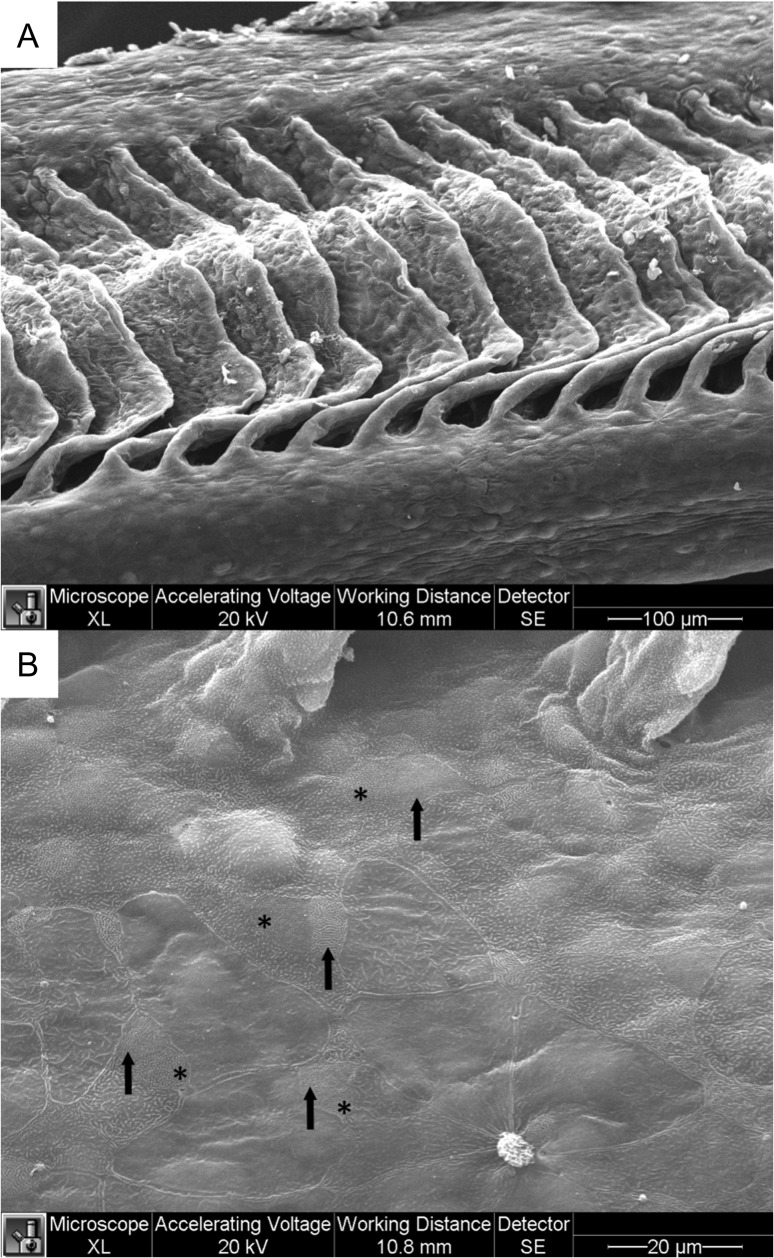
Scanning electron micrograph of Arctic grayling gills in freshwater. (**A**) Single gill filament with exposed protruding lamellae. (**B**) Higher magnification of trailing edge of filament near base of protruding lamellae. Apical surface reveals large flat pavement cells or covered with shortened unconnected microplicae, along with two types of MR cells similar to that of trout interspersed among pavement cells, one type with finger-like projections marked with asterisks (*) and another type with tightly packed microvilli forming with a maze-like appearance indicated by arrows (↑).

**Figure 7:**
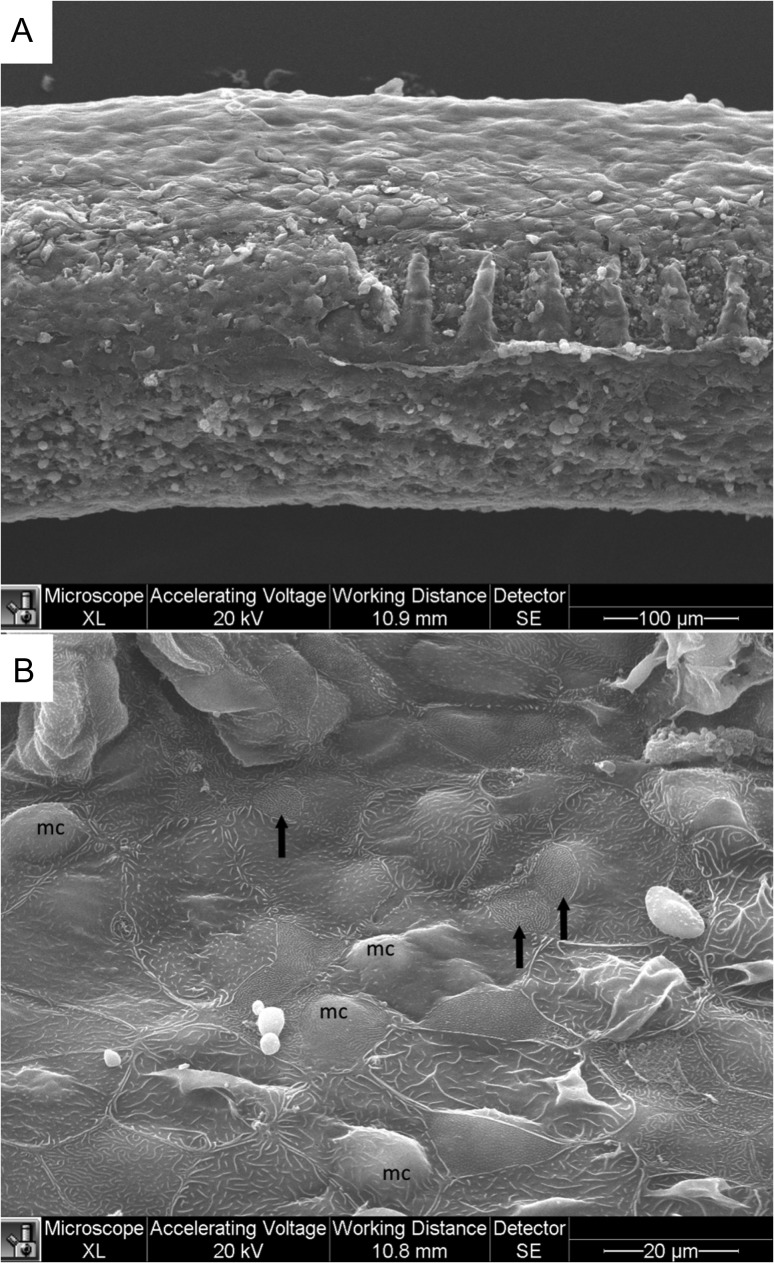
Scanning electron micrograph of Arctic grayling gills following 24 h exposure to 17 ppt salinity. (**A**) Single gill filament with limited number of exposed lamellae and increased ILCM. (**B**) Higher magnification of trailing edge of filament near base of protruding lamellae. Apical surface of the trailing edge reveals large protruding mucous cells (mc) as well as a dehydrated like appearance of apical surface of pavement cells. Some MR cells (arrow) still apparent on trailing edge of filament; however, 006no apical crypt development was seen.

In comparison to the structure of the rainbow trout gill, the Arctic grayling filament was composed of lamellae in similar branching orientation, however, they extended laterally to a lesser extent leaving more surface area on the leading and trailing edges (Fig. 6A). Large epithelial pavement cells covered the filament with their apical surfaces either flat or ridged with shortened microplicae in a less continuous fashion than those of the trout (Fig. 6B). Differentiated MR cell types commonly appeared in pairs and their surface characteristics were almost identical to those of trout with one type having a more dotted appearance of short finger-like microvilli, while the other surface had a compact maze-like quality (Fig. 6B). Exposure to salinity resulted in reduced lamellar surface area, with the ILCM dominating (Fig. 7A). Swelling mucous cells protruded from the Arctic grayling epithelium and were more prevalent upon exposure to 17 ppt. The gill epithelium of the Arctic grayling did not exhibit any apical crypt formation on the MR cells in response to salinity exposure, which is characteristic of salinity acclimation in the more euryhaline rainbow trout (Fig. 7B).

**Figure 8:**
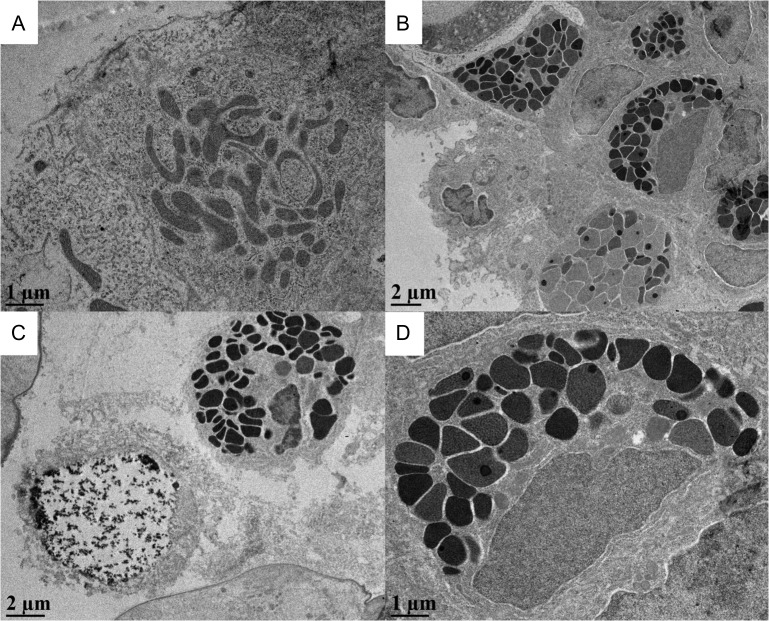
High magnification of various cell types found within the Arctic grayling ILCM. (**A**) Mitochondria rich cell, (**B**) granulocytes/eosinophils and apoptotic cells, (**C**) granulocyte and bursting/releasing cell and (**D**) enhanced granulocyte.

Transmission electron micrographs of Arctic grayling gill sections revealed the presence of different cell types near the apical surface depending on the environmental salinity, as well as the various cell types present in the interlamellar cell mass. Figure 8 depicts high magnifications of the various cell types (mitochondrion rich cells, granulocytes/eosinophils and bursting/releasing cells) and internal organelles present in the ILCM of Arctic grayling.

**Figure 9:**
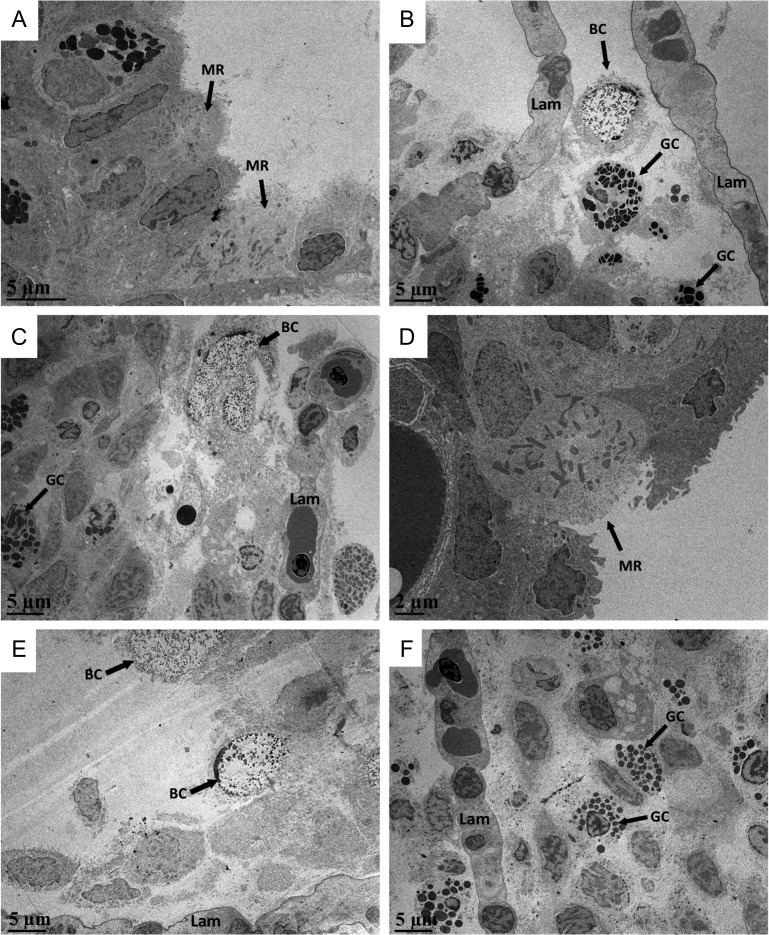
Transmission electron micrographs of Arctic grayling ILCM under various salinity exposures. (**A**) FW gill interlamellar space, with MR cells and granulocytes (black granules), (**B**) 12 h salinity exposure with elevated ILCM containing granulocytes and apparent bursting/releasing cells on apical surface, lacking MR cells on apical surface, (**C**) 24 h salinity exposure with granulocytes and bursting/releasing cells, (**D**) Trailing edge of gill following 24 h recovery, with exposed MR cell, (**E**) 48 h exposed ILCM with apically located bursting/releasing cell, (**F**) 48 h plus recovery, ILCM still slightly elevated with many granulocytes present and other cells containing irregular shaped nucleus appear to be undergoing apoptosis. Lam, lamellae; MR, mitochondrion rich cell; GC, granulocyte; BC, bursting/releasing cell.

Representative samples of control fish (no exposure to higher salinities) showed both exposed mitochondria rich cells, mucous cells and some eosinophil-like cells (containing large granulocytes) at the apical surface of the filament in the interlamellar space (Fig. 9A). Upon exposure to 17 ppt at 12 h and persisting through 48 h, the elevated interlamellar cell mass was identifiable and was characterized by an increased number of mucous cells throughout and near the surface of the elevated ILCM, as well as what appeared to be eosinophils bursting or releasing their granular material (Fig. 9B). These bursting cells were not present in any freshwater samples. Furthermore, MR cells were not found in the interlamellar cell mass in any of the salinity exposed samples. Representative micrographs of gills from fish recovering in freshwater for 24 h post-salinity exposures, displayed the ILCM dominated by mucous cells, as well as non-descript dead cells, near the surface of the ILCM (as indicated by the denatured formation of the nucleus). However, on the edges of the filament (trailing edge), the surface contained healthy MR cells and mucous cells (Fig. 9C–E). The 48 h exposure plus recovery samples were characterized by apparent apoptotic cells with irregular nuclei and increased number of granulocytes/eosinophils (Fig. 9F).

## Discussion

The present study was performed to understand the unique and important physiological responses to industrially relevant environmental disturbances in the sensitive native salmonid species, the Arctic grayling. Physiological data confirm the poor salinity tolerance and relatively stenohaline behaviour of this salmonid while novel morphological evidence effectively provides insight into the cell and tissue alterations occurring during these osmotic perturbations. Our study answers the significant ecological and conservation science-relevant question of whether grayling are capable of recovery if re-introduced into freshwater following a short-term environmental exposure to high salinity. In addition to characterizing the gill remodelling that occurs following exposure and recovery from acute elevated environmental salinity, this is also the first study to provide general branchial morphological characteristics of the Arctic grayling.

As we reported earlier (Blair *et al.*, 2016), changes in serum levels of ions and osmolality occur in both grayling and trout within 24 h of acute exposure to 17 ppt salinity. Significant and increasing elevations in these levels through 48 h post-exposure indicate osmotic imbalances and physiological osmoregulatory mechanisms must be activated to alleviate this stress and lower the plasma salt concentrations. In most salmonids, a return to a homoeostatic level usually occurs within 4 days following salinity exposure, (Finstad *et al.*, 1988; McCormick *et al.*, 1989; Richards *et al.*, 2003; Bystriansky *et al.*, 2006). However, Arctic grayling cannot successfully osmoregulate upon 17 ppt exposure, displaying serum osmolality and ionic concentrations highly elevated above those of the tolerant rainbow trout, with the grayling becoming moribund around 96–100 h of exposure (Blair *et al.*, 2016). However, if grayling are allowed to return to freshwater, even after 48 h, they recover serum ion and osmolality levels by 24 h.

The presence of an ILCM was previously shown to appear upon 24 h exposure to 17 ppt salinity. In this study, we have demonstrated that the ILCM is beginning to form even at 12 h post-exposure. This rapid gill remodelling of increasing ILCM is one of the fastest reported to date, with other reports occurring at one week of air exposure in *Kryptolebias marmoratus* (*Ong et al.*, 2007); hyperplasia occurring at 8 days in rock bass, largemouth bass and black crappie exposed to pH 4.0 and first observed at 2 days for yellow perch (McCormick *et al.*, 1989), and at 4 days in brook trout acclimated to pH 5.2 and aluminium (Mueller *et al.*, 1991); although decrease of ICLM was also reported within 1 day in goldfish exposed to hypoxia (Mitrovic *et al.*, 2009). Again, when Arctic grayling are allowed to recover in freshwater for 24 h, ICLM decreased in comparison to the 24 and 48 h exposures to 17 ppt salinity. This demonstrates the ability for rapid reduction of the ILCM when favourable conditions return, which would allow for increased surface gill area for oxygen uptake and salt excretion.

We hypothesized that ILCM development provides a temporary defence mechanism and that this gill plasticity is a mechanism to protect these fish against environmental perturbations. Sollid *et al.* (2003) similarly demonstrated this ability in crucian carp gills that normally lack protruding lamellae under normoxic conditions. However, when exposed to hypoxia, the interlamellar cell mass begins to decrease thus exposing the secondary lamellae which greatly increases the surface area for oxygen uptake from the environment. This gill remodelling was associated with a lack of cell mitosis and an increase in apoptotic cells within the ILCM under hypoxia conditions (Sollid *et al.*, 2003). We are unsure of the mechanisms required for shedding of the ILCM in Arctic grayling, but future investigations into the mechanisms allowing rapid formation and reduction of the ILCM should be investigated.

The gill is the primary site of ionoregulation with specific ion transporters expressed on the apical surface of mitochondria rich cells (Dymowska *et al.*, 2012). Our SEM and TEM results reveal that during salinity exposure, the ILCM area is characterized by a lack of MR cells on the apical surface suggesting an impairment of ionoregulatory ability. This contrasts with others who found MR cells on the apical surface of the ILCM of crucian carp acclimated to 7°C (Mitrovic and Perry, 2009). The reasons for the distinction are unknown but we suggest that crucian carp slowly develop (over 7 days) an ILCM as a natural process to allow long-term survival under ice in the winter. Given the long-term nature of the responses, the animal would need to include MR cell activity for ion and acid–base regulating capacity. However, in grayling, the ILCM formed in response to an abrupt ionoregulatory disturbance and the presence of ion transporters on the surface might exacerbate ion loading by driving transport processes in reverse to load the animal with salts. Clearly, given the broad nature of ILCM formation in a variety of fish species, it is necessary to understand the proximal mechanism and physiological responses of each individual case.

The increased ILCM is composed of undifferentiated cells, pavement cells, mucous cells and granulocyte containing cells resembling eosinophils. The presence of MR cells was still apparent on the trailing edge of the filament, which likely allowed for the maintenance of basal ion transport during the increased ILCM event. The appearance of the granulocyte cells and mucous cells is indicative of gill irritation or stress, and we surmise, based on the increased presence of eosinophils, is the result of a localized immunologic response. Just as mast cells are mobilized and trafficked into the gill epithelium in the presence of a parasite (Dezfuli and Giari, 2008), the gills of fish may also undergo similar influx of leucocytes following immune stimulation from an environmental irritant, in this case salinity. Reite (1997) provides corroborative findings demonstrating an induced bacterial infection resulted in inflammation and hyperplasia in the gill epithelium of brown trout, brook trout and European grayling. Significantly, the authors noted the recruitment of large numbers of granule cells associated with this gill epithelium inflammation (Reite, 1997). We suggest that a similar immune response could be present in the gills of Arctic grayling and this could play a role in the development of an ILCM following hyperosmotic stress.

In the event of a spill of hypersaline-produced water, the osmotic perturbations to the aquatic organisms present a real threat and fish kills have been documented following spills. For example, in 2007, hydraulic fracturing fluids overflowed a retention pond and spilled into Acorn Fork Creek (Knox County, Kentucky) raising stream conductivity from 200 μS/cm (<1 ppt) to over 35 000 μS/cm (>25 ppt) (Papoulias and Velasco, 2013). High salinity along with low pH resulted in death or displacement of many aquatic invertebrates and fish including the threatened Blackside Dace, *Chrosomus cumberlandensis*, which much like the Arctic grayling, need cool clear water to thrive. Other affected fish included creek chub, which demonstrated extreme gill lamellar hyperplasia (we refer to as increased ILCM) which in this study was associated with a combination of effects resulting from decreased pH, metal toxicity and increased conductivity (salinity) (Papoulias and Velasco, 2013). While these results are quite similar to the changes seen in the present study, we have demonstrated the gill remodelling can be elicited by salinity alone in this population of Arctic grayling. This may have implications for other studies associated with highly saline waters from hydraulic fracturing spills including the interpretation of results and consistent need for salt controls during physiological experiments.

It is extremely important to note volume, concentration and time of a spill when interpreting the imminent negative consequences to the environment and organisms. As our study demonstrates, if spills result in environmental salinity increasing to <17 ppt, Arctic grayling can potentially survive this perturbation if the salinity stress is removed within 48 h. It can be theorized that under environmental spill conditions an ‘acute transfer’ of freshwater organisms into salinity can indeed occur given a large sudden spill into a river, although a slower transition is more likely under the circumstances of a pipeline break or overland leak of smaller relative volume. In turn, the rapid recovery to freshwater could also be feasible given the leak was quickly fixed or flow was stopped. However, a protracted recovery to full freshwater is more likely, and a slower recovery of plasma ion levels as well as a reduction ILCM would reflect that transition.

Our fresh waters are at a constant risk of increasing salinity due to anthropogenic forces including, road salt application, hydraulic fracturing and other oil and gas activities, and decreasing flows due to climate change. Salinity is known to have adverse consequences on the aquatic ecosystem. For this reason, increased regulations and higher standards need to be met and enforced regarding salinity effects on freshwater environments (Cañedo-Argüelles *et al.*, 2016). Currently, the rainbow trout is used as a regulatory species for effluent testing and acute toxicity (USEPA, 2002). However, it may be necessary in some situations for regulations to be based on the local sensitive species near potential spill areas rather than the rainbow trout, which demonstrates higher salinity tolerance. Without discounting this clear obligation, many of our freshwater species are indeed able to cope with minor osmotic changes or perturbations which plague their habitat, within a certain species-specific tolerance range. The Arctic grayling maintains the ability to develop a potentially protective interlamellar cell mass in order to alleviate these stresses to a certain threshold. This ILCM seems to be associated with some type of immune response indicated by the presence of granulocyte containing cells within the resulting mass. Furthermore, upon the return of favourable conditions, the grayling blood serum ion levels decreased along with marked reductions in the ILCM. Further research into the exact mechanisms behind the development and reduction of the ILCM is necessary.
